# Staff-reported barriers and facilitators to the implementation of healthcare interventions within regional and rural areas: a rapid review

**DOI:** 10.1186/s12913-025-12480-8

**Published:** 2025-03-04

**Authors:** Anna Chapman, Alison Buccheri, Devdini Mohotti, Anna Wong Shee, Catherine E. Huggins, Laura Alston, Alison M. Hutchinson, Sze Lin Yoong, Hannah Beks, Kevin Mc Namara, Anna Peeters, Anna Ugalde

**Affiliations:** 1 Centre for Quality and Patient Safety Research, Institute for Health Transformation, School of Nursing & Midwifery, Faculty of Health, Deakin University, Building Y, 221 Burwood Hwy, Burwood, VIC 3125 Australia; 2 Research Unit, Colac Area Health, Colac, VIC Australia; 3 Deakin Rural Health, Faculty of Health, Deakin University, Warrnambool, VIC Australia; 4 Grampians Health, Ballarat, VIC Australia; 5 Global Centre for Preventive Health and Nutrition, Institute for Health Transformation, School of Health and Social Development, Faculty of Health, Deakin University, Geelong, VIC Australia; 6 Barwon Health, Geelong, VIC Australia; 7 Institute for Health Transformation, Faculty of Health, Deakin University, Geelong, VIC Australia

**Keywords:** Rural health, Implementation science, Barriers, Facilitators, Review, Healthcare

## Abstract

**Background:**

Individuals in rural areas consistently demonstrate higher mortality and morbidity rates, and poorer access to healthcare, compared to their metropolitan counterparts. Optimizing the implementation of evidence-based interventions can reduce these inequities. Existing literature outlines numerous barriers and facilitators to the implementation of healthcare interventions, but these are generally not specific to rural areas. This rapid review aims to synthesize barriers and facilitators to the implementation of healthcare interventions in regional and rural healthcare services as reported by healthcare staff, including clinicians, managers, and administrators.

**Methods:**

A systematic search for peer-reviewed publications was conducted using CINAHL, PsycINFO, Medline, and Embase databases (1/1/2000–29/08/2023). Eligible publications were primary research articles published in English, assessing staff-reported barriers and facilitators to implementing healthcare interventions within regional and rural areas of high-income countries. Qualitative, quantitative, and mixed-methods designs were included. Eligible healthcare settings encompassed acute, sub-acute, primary care, community health, and aged care. Barrier and facilitator data were coded and grouped into sub-themes and broader themes, with results presented narratively.

**Results:**

Thirty-nine publications met the inclusion criteria. Most studies were conducted in Australia or the USA (both *n* = 18, 46%), within primary care (*n* = 13, 33%) or hospital settings (*n* = 12, 31%) in rural (*n* = 22, 56%) or regional (*n* = 9, 23%) locations. Implementation barriers and facilitators were grouped into four overarching themes: intervention-level (intervention feasibility and fit; complexity; privacy and confidentiality); staff-level (staff attitudes and beliefs; knowledge, skills, and confidence; staff roles and professional identity), patient-level (patient characteristics; attitudes), and system-level (leadership support; environmental resources and context; geographic vastness; networks and communication).

**Conclusions:**

These findings provide essential guidance for policymakers, healthcare leaders, and researchers in planning and designing future implementation efforts in regional and rural healthcare settings. By considering factors across intervention, staff, patient, and system levels, stakeholders can address challenges and leverage local strengths to enhance implementation success and reduce health disparities.

**Trial registration:**

PROSPERO registration number: CRD42023470736. Registered 19/10/2023.

**Supplementary Information:**

The online version contains supplementary material available at 10.1186/s12913-025-12480-8.

## Background

The persistent gap in health outcomes between rural and metropolitan areas represents a significant challenge for healthcare systems globally [1]. Individuals living in rural areas, which are generally characterized by geographical vastness and sparse population distribution [2], consistently experience higher mortality and morbidity rates, along with poorer access to healthcare services, compared to their metropolitan counterparts [3, 4]. These disparities in provision and access of care are influenced by a complex interplay of social, economic, geographical, and system-related factors [5].

Optimizing the implementation of evidence-based interventions (EBIs) has emerged as vital to addressing the health inequities experienced by key population groups, such as those living in regional and rural areas [6]. EBIs are broadly defined as practices, programs, policies, processes, or guideline recommendations that have been proven effective in improving health outcomes [7]. When implemented as intended, EBIs have significant potential to enhance the quality of care and outcomes for people living in regional and rural settings [8]. Yet, integrating EBIs into routine healthcare practice is challenging and complex, with many healthcare interventions not routinely adopted, delivered or sustained [9].

To support the uptake and sustainability of EBIs, the identification of context-specific barriers and facilitators (i.e. determinants) to implementation is recommended as an important step that enables the design of tailored, theory-informed implementation strategies [10]. While existing implementation research has extensively documented various determinants to implementation across a range of healthcare contexts [11–13], there remains a significant gap in understanding the unique experiences of regional and rural areas. Healthcare services in these areas are required to leverage strengths and overcome challenges such as geographic spread, low population density, limited infrastructure, and workforce retention to effectively implement EBIs [14]. A nuanced understanding of the factors influencing implementation in regional and rural healthcare services will be essential for bridging the health disparities between rural and metropolitan populations.

To address this gap, we aimed to synthesize barriers and facilitators to the implementation of healthcare interventions in regional and rural healthcare services as reported by healthcare staff, including clinicians, managers, and administrators. Given the significant differences between high- and low-income healthcare settings [15]—such as resources, infrastructure, health system structure, workforce, disease burden, and policy environments—this rapid review specifically focused on healthcare staff working in high-income countries. Healthcare staff are uniquely positioned to provide valuable insights into the practical realities of implementing healthcare interventions in their settings. By focusing on their lived experiences, this review will inform future implementation efforts in regional and rural areas and contribute to the design of more effective and equitable healthcare solutions.

## Methods

### Design

This study adopted a rapid review design, a form of knowledge synthesis that accelerates the process of conducting a traditional systematic review through streamlining or omitting various methods to produce evidence for stakeholders in a resource-efficient manner [16]. This approach met the practical need of informing the authors’ broader research program, funded by the Medical Research Future Fund in Australia under the Rapid Applied Research Translation initiative. In this review, streamlining of methods included applying limiters to the systematic search (English-language, year of publication 2000 +), using single data extraction (checked for accuracy), and omitting the critical appraisal step. Additionally, knowledge users, specifically clinicians based in regional and rural areas, were included as part of the authorship team, and participated in all stages of the review process. The Preferred Reporting Items for Systematic reviews and Meta-Analysis (PRISMA) [17] statement and the interim guidance for the reporting of rapid reviews [18] guided the reporting of this review (See Supplementary File 1); the protocol was prospectively registered in PROSPERO (CRD42023470736).

### Search strategy

A literature search was conducted on 29/08/2023 using four electronic databases (Medline, Embase, PsycINFO and CINAHL); additional studies were identified by searching the reference lists of included papers. The search was limited to articles published after 1 January 2000, aligning with the emergence of implementation science as a distinct discipline [19]. While healthcare interventions have advanced rapidly in the past decade, this broader timeframe ensured the inclusion of both foundational implementation research and recent developmentsrelevant to contemporary healthcare practices. Preliminary literature searches and key terms from relevant literature [11, 20] guided the development of a comprehensive list of search terms. The search strategy used key terms and synonyms related to the following concepts: the regional/rural context, implementation, healthcare setting, barriers and facilitators (Supplementary File 2). To increase the sensitivity of the search, key terms that mapped to specific subject headings (e.g., MeSH) were included and adapted according to each database. Additionally, truncations and expansions were applied to selected search terms.

### Inclusion and exclusion criteria

A summary of the eligibility criteria for this review is provided in Table 1. Barriers and facilitators were broadly conceptualized as any factor that either hindered or promoted the implementation process of healthcare interventions and included synonymous terms, such as challenges and enablers. The designations of factors as barriers and facilitators within primary research studies were accepted as reported. Classifications of regional and rural were also accepted as reported; authors did not have to explicitly refer to a geographical classification system (e.g., Rural–Urban Continuum Codes (RUCC) [21] or Modified Monash Model [22] (MMM)) for a study to be considered eligible, although this information was extracted from included papers. Inclusion was limited to studies conducted in high-income countries, as defined by the World Bank classification [23] (as at 11 August 2023). Importantly, papers that only assessed intervention effectiveness e.g., clinical or health outcomes, rather than factors influencing the implementation process, were excluded.

**Table 1:** Summary of eligibility criteria for the review

Criteria	Included	Excluded
Population	Healthcare staff in eligible context. Includes clinicians, managers, and administrators	Non-healthcare staff, or healthcare staff based in an ineligible context
Context	Acute, sub-acute, primary, community health, and residential aged care settings	Non-healthcare settings (e.g., workplaces, schools)
	Regional and rural areas (e.g., MMM 2–5)	Major cities/metropolitan areas (e.g. MMM 1) & remote areas (e.g., MMM 6–7); with regional and/or rural areas not separated in analysis
	High-income countries, according to The World Bank rankings [23] (excluded at full-text stage only)	Low- and middle-income countries (excluded at full-text stage only)
Intervention of focus	Healthcare interventions encompassing direct patient interventions (e.g., clinical or therapeutic practices or programs) or interventions with an intended benefit on health outcomes or quality of care (e.g., guidelines, processes, models of care)	Non-healthcare interventions/interventions with a primary focus on administrative outcomes, rather than quality of care or health outcomes. (e.g., management interventions, staff rostering interventions, referral only interventions)
Outcomes	Healthcare staff-reported barriers and facilitators to the implementation process. Could be collected during any stage of implementation (i.e., pre-implementation, during implementation, or post-implementation)	Not healthcare staff-reported barriers and facilitators to implementation (i.e., patient-reported barriers)
Publication type	Original research, including case-studies, qualitative, quantitative, and mixed method research	Theses, conference abstracts, reviews, commentaries, editorials, published books, protocols
	English language full-text articles only	Articles not published in English
Publication date	Published from 1 January 2000-August 29 2023	Published prior to 1 January 2000

### Study selection and data analysis

Database search results were imported into Endnote X9 to remove duplicates; remaining records were then uploaded to Covidence. All records were independently screened in duplicate by title and abstract; potentially relevant full-text articles were then dual-screened against pre-specified eligibility criteria. Any screening conflicts were resolved through discussion or by involving the senior author. Only full-text articles that met all inclusion criteria progressed to data extraction.

Data extraction was performed using REDCap, with each paper undergoing single data extraction. A broad team of reviewers extracted data on study characteristics, including year and country of study, description of rurality, participant characteristics, description of intervention, and methodological approaches. Two authors (AC & DM) concurrently extracted barrier and facilitator data; these data were extracted in narrative form as per the explicit reporting within each article. Following single extraction, the first author reviewed data for completeness.

A narrative synthesis approach was employed to integrate and interpret the extracted barrier and facilitator data [24]. Relevant data fields were exported from REDCap into Excel and organized into a summary table that included the name, description, illustrative quotes, and the designation of whether each item was categorized as a barrier or a facilitator within each study. This summary table was then imported into NVivo software, where two authors (AC & AB) independently coded data, applying an inductive approach to identify key concepts. These concepts were first organized into sub-themes and then grouped into overarching themes. Given most included studies did not formally use an implementation framework to collect, analyze, or present their findings, data were not coded according to a specific implementation framework. To enhance the rigor of synthesis, themes and sub-themes were discussed among the broader author team, allowing the incorporation of diverse perspectives, including input from clinicians with lived experience in implementing healthcare interventions in rural and regional areas. In addition to the narrative synthesis, summary statistics were generated to describe the study characteristics.

## Results

### Search results

**Fig. 1:**
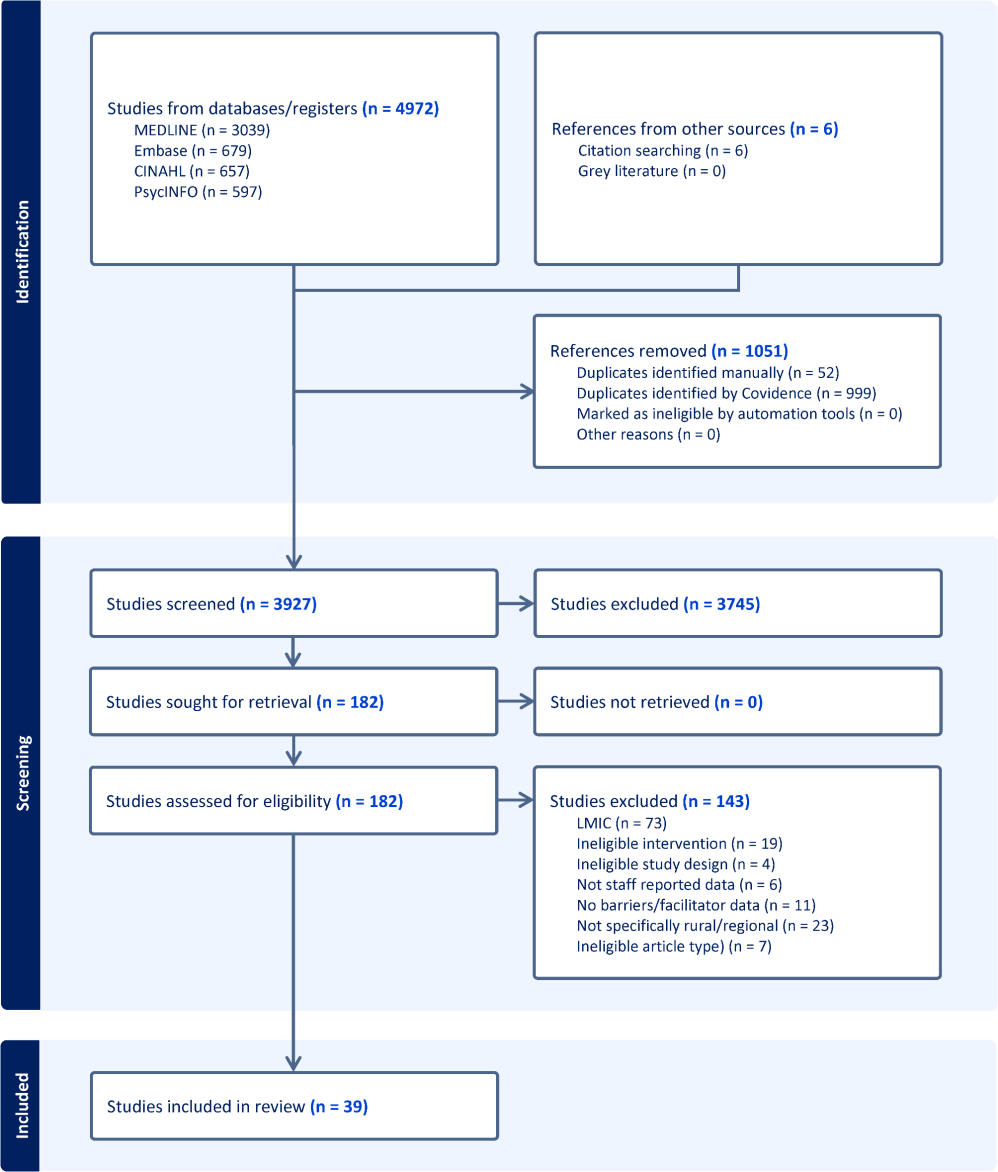
PRISMA Flow chart

After removing duplicates, the search yielded 3,927 citations, from which 182 full texts were examined. Ultimately, 39 publications met the inclusion criteria. The PRISMA flow diagram details the search process and reasons for exclusion (Fig. 1).

### Characteristics of included studies

Characteristics of included studies are provided in Table 2. Most studies were published within the previous five years (2019–2023: *n* = 27, 69%) and were conducted in Australia or the United States of America (USA) (*n* = 18 each, 46%). The majority described their context as rural (*n* = 22, 56%), followed by regional (*n* = 9, 23%) and mixed rural/regional (*n* = 8, 21%). Only seven (18%) studies explicitly used a geographical classification system to define the context (Australian Statistical Geography Standard: *n* = 2, Urban Influence Codes: *n* = 2, MMM *n* = 1, Accessibility/Remoteness Index of Australia *n* = 1, RUCC *n* = 1). Primary care (*n* = 13, 33%), hospital (*n* = 12, 31%), and mixed healthcare settings (*n* = 8, 21%) were the most common healthcare settings, followed by community health (*n* = 3, 8%), and residential aged care (*n* = 3, 8%).

**Table 2:** Summary table of included articles (*n* = 39)

First author, citation	Year	Country	Health service	Intervention of focus (classification type)	Staff sample characteristics	Rurality	Data collection approach^b^	Use of implementation TMF
Andrilla [25]	2017	USA	Primary care	Buprenorphine maintenance treatment– prescription of buprenorphine to treat opioid use disorder. (Clinical Practice/ Program)	(*N* = *1,124*). Physicians	Rural, as defined by an Urban Influence Code designation	Quantitative (surveys)	No
Beks [26]	2022	Australia	Community health	Tulku wan Wininn primary health mobile clinic using an Aboriginal Community-Controlled Health Organization model of service delivery (Model of Care)	*(N* = *19).* Included health service personnel and key informants (*n* = 12), and Aboriginal and/or Torres Strait Islander clients (*n* = 7)	Rural, as defined by the Modified Monash Model	Qualitative (interviews)	No
Berends [27]	2012	Australia	Hospital	Screening and brief intervention for risky alcohol use and referral to treatment (Screening & Assessment)	*(N* = *149)*. Survey participants (nurses; *n* = 142); Interview participants (stakeholders; *n* = 7)	Regional^a^	Mixed methods (surveys, interviews)	No
Chatterton [28]	2022	Australia	Community health, public mental health service with tertiary education provider	Telehealth mental health response in a regional public mental health provider. Targeted to healthcare providers and patients (Digital Health Intervention)	(*N* = *32*). Medical, nursing and allied health staff	Regional^a^	Qualitative (interviews, focus groups)	No
Daugherty [29]	2021	USA	Hospital, Primary care	Clinical Recommendations in the CDC Paediatric Mild Traumatic Brain Injury Guideline– targeted to healthcare providers and patients (Guideline/ Recommendation)	(*N* = *9*). Healthcare providers, including physicians (*n* = 5); physician assistant (*n* = 2); nurse practitioners (*n* = 2)	Rural^a^	Qualitative (interviews)	No
DeHart [30]	2021	USA	Hospital, Primary care	Telehealth modalities including live videoconferencing, store-and-forward, remote patient monitoring and mobile health (Digital Health Intervention)	(*N* = *17*). Health providers, including administrators (*n* = 7); nurses (*n* = 3); physicians (*n* = 2); dieticians (*n* = 2); counsellors (*n* = 1); social workers (*n* = 1); educator (*n* = 1)	Mixed rural/regional^a^	Mixed methods (surveys, interviews, focus groups)	No
Delaforce [31]	2023	Australia	Hospital	Concentric Care fall prevention platform– digital intervention targeted to nursing staff in inpatient settings. Consists of speech-enabled nurse call system, location services and digital dashboards to assist with care provision for high-risk fall patients (Digital Health Intervention)	(*N* = *12*). Nursing staff, including nurses (*n* = 8); nurse managers (*n* = 4)	Rural^a^	Qualitative (interviews, focus groups)	Yes (CFIR)
Druskin [32]	2022	USA	Range of healthcare settings	Parent–Child Interaction Therapy targeted to patients, families, and healthcare providers– opioid crisis-related emotional and behavioural intervention for children, teaches positive and appropriate strategies for parents. (Clinical Practice/ Program)	(*N* = *34*). Therapists at doctoral level and masters level, and doctoral student trainees	Rural^a^	Qualitative (interviews)	No
Dwyer [33]	2020	Australia	Hospital	Australian Commission on Safety and Quality in Health Care's Acute Stroke Clinical Care Standard (Guideline/ Recommendation)	(*N* = *11*). Clinicians; including interview participants (*n* = 3; pharmacist, senior nurse, physiotherapist); focus group participants (*n* = 8; neurologists, a general medical physician)	Regional, using the Accessibility/Remoteness Index for Australia 2011 system	Qualitative (interviews, focus groups)	No
Ervin [34]	2019	Australia	RAC	Older person's nurse practitioner role and extended scope of practice in residential aged care (Model of Care)	(*N* = *58*). Health service management, care managers, nursing and care staff, and GPs	Mixed rural/regional^a^	Qualitative (interviews, focus groups)	Yes (May’s Implementation Theory)
Findholt [35]	2013	USA	Primary care	Implementation of the American Medical Association Expert Committee recommendations for assessment, treatment and prevention of childhood obesity (Guideline/ Recommendation)	(*N* = *13*). Clinicians	Rural, using Urban Influence Code designations	Qualitative (interviews)	No
Fletcher [36]	2016	Australia	Hospital, primary care	Advanced Care Planning– discussion exploring issues of end-of-life experience for patients in aged care facilities. Targeted to healthcare providers and patients (Care Planning)	(*N* = *55*). GPs, general practice registrars, practice nurses, community nurses, hospital nurses	Mixed rural/regional, as defined by ASGC– Remoteness Area	Qualitative (focus groups)	No
Harrod [37]	2014	USA	Hospital	Effort to prevent healthcare-associated infections for patients—included data on the local safety culture, administrative support for infection prevention, and hospital's infection prevention program (Clinical Practice/ Program)	(*N* = *72*). Lead infection preventionists at rural veterans’ affairs hospitals	Rural^a^	Mixed methods (surveys, interviews)	No
Henderson [38]	2018	Australia	Range of healthcare settings	Integrated care for older people with mental health problems (Model of Care)	(*N* = *31*). Healthcare providers; included NGO (*n* = 3); social support services (*n* = 3); mental health (*n* = 4); aged care (*n* = 4); primary care (*n* = 9); community health (*n* = 6); local government (*n* = 1); hospital (*n* = 1)	Mixed rural/regional^a^	Qualitative (interviews)	No
Hill [39]	2022	Australia	RAC	Provision of prescribed texture-modified food and fluids for residents with dysphagia (Guideline/ Recommendation)	(*N* = *11*). Included nurses (*n* = 6) and food services staff (*n* = 5) in aged care facility	Rural^a^	Qualitative (focus groups)	No
Howland [40]	2021	USA	Primary care	Telepsychiatric Collaborative Care (support to providers treating patients at FQHCs) or telemental health referral (videoconferencing patient assessment and treatment at FQHC) (Digital Health Intervention)	*(N* = *14).* Including off-site telepsychiatrists (*n* = 10); telepsychologists (*n* = 4)	Rural^a^	Qualitative (interviews)	Yes (CFIR)
Khoong [41]	2014	USA	Primary Care	Clinical preventive service guidelines (Guideline/ Recommendations)	(*N* = *29*). Primary care physicians	Mixed rural/regional^a^	Qualitative (interviews)	No
Kilcullen [42]	2017	Australia	Hospital	Delivery of neonatal palliative care by neonatal ICU nurses (Clinical Practice/ Program)	(*N* = *8*). Neonatal ICU nurses with experience providing neonatal palliative care	Mixed rural/regional^a^	Qualitative (interviews)	No
Kirstman-Valente [43]	2022	USA	Primary care	Cannabis use reduction screening, brief intervention, and referral to treatment model for adolescent cannabis users (Model of Care)	(*N* = *11*). Patient-engaged providers/staff from Seattle Children’s Care Network Includes paediatricians (*n* = 7); paediatric nurse practitioners (*n* = 2); registered nurse (*n* = 1); practice manager (*n* = 1)	Regional^a^	Qualitative (focus groups)	No
Kruse-Diehr [44]	2022	USA	Primary care	Colorectal Cancer Screening (Screening & Assessment)	(*N* = *7*). Medical providers, primarily nurses from 4 primary care clinics	Rural^a^	Qualitative (interviews)	No
Lam [45]	2018	Australia	RAC	Advanced Care Planning– discussion exploring issues of end-of-life experience for patients in aged care facilities (Care Planning)	(*N* = *114*). Nurses, care managers, occupational therapists, medical doctors. Includes survey participants (*n* = 109; interview participants (*n* = 5)	Rural^a^	Mixed methods (surveys, interviews)	No
Lillebuen [46]	2020	Canada	Hospital	Peritoneal dialysis provided by ED nurses (Clinical Practice/ Program)	(*N* = *7*). Nurse managers and clinical nurse educators employed at participating sites	Rural^a^	Qualitative (interviews)	No
Littlewood [47]	2019	Australia	Primary care	Advanced Care Planning—supporting adults to understand and share values, goals, and future medical care preferences during serious and chronic illnesses (Care Planning)	(*N* = *13*). General practice registrars and recently fellowed GPs	Rural, as defined by the Australian Statistical Georgraphical Classification– Remoteness Area	Qualitative (interviews)	No
Maxwell [48]	2021	Australia	Community health	Reablement-focused program for older people in regional Australia. Intervention included training for community-based organisational staff to incorporate and reflect on reablement into practice (Clinical Practice/ Program)	*(N* = *17).* Included care coordinators (*n* = 13, focus groups); direct care staff (*n* = 4, interviews)	Regional^a^	Qualitative (interviews, focus groups)	No
Morgan [49]	2019	Canada	Primary care	Evidence-based interdisciplinary primary healthcare memory clinic for dementia (Model of Care)	*(N* = *25)*. Included primary healthcare team members, managers, office staff	Rural^a^	Qualitative (interviews, focus groups)	Yes (CFIR)
Muir-Cochrane [50]	2014	Australia	Community health, RAC	Mental health care for older people through health and social services (Clinical Practice/ Program)	(*N* = *19*). Key informants from 16 local government and non-government health and social care agencies	Rural^a^	Qualitative (interviews)	No
Nelson-Brantley [51]	2021	USA	Primary care	Cancer screening practices in rural primary care practices (Screening & Assessment)	(*N* = *28*). Physicians, nurses, osteopath, medical assistant, care coordinator and administrators in primary care practices	Rural, as defined by Rural–Urban Continuum Codes	Qualitative (focus groups)	Yes (CFIR)
Paliadelis [52]	2005	Australia	Hospital	'Family centred care'– paediatric nurses’ involvement of parents in the care of hospitalized children (Model of Care)	(*N* = *14*). Paediatric nurses	Regional^a^	Qualitative (interviews)	No
Parchman [53]	2020	USA	Primary care	The Six Building Blocks program—management of patients using long-term opioid therapy. Includes leadership support, revision and alignment of policies, tracking and monitoring patients, planned, patient-centred visits, identifying and connecting to resources for complex patients, and measuring success (Model of Care)	(*N not specified).* Staff and clinicians, including opiod improvement team members, from six organisations	Rural^a^	Qualitative (interviews, focus groups)	No
Porter [54]	2021	USA	Primary care	Three evidence-based intensive weight management programs: (1) Calcium Weighs-In (2) TOURS, and (3) diaBEAT-it! (Clinical Practice/ Program)	*(N* = *51).* Included physicians (*n* = 15); nurses (*n* = 11); clinic managers/ administrators (*n* = 9); physical assistants/nurse practitioners (*n* = 7), clinic staff (*n* = 7); health coach /coordinator (*n* = 2)	Mixed rural/regional^a^	Qualitative (focus groups)	Yes (PARiHS Framework)
Rosado [55]	2023	USA	Primary care	Adverse childhood experiences screening protocol (Screening & Assessment)	(*N* = *12*). Paediatricians; survey participants (*n* = 6); focus group participants (*n* = 6)	Rural^a^	Mixed methods (surveys, focus groups)	Yes (EPIS Framework)
Rosenberg [56]	2022	USA	Hospital	ED-initiated buprenorphine for patients presenting with OUD (Clinical Practice/ Program)	(*N* = *11*). ED Directors from Critical Access Hospital EDs	Rural^a^	Qualitative (interviews)	No
Saunders [57]	2019	USA	Primary care	Substance use screening (alcohol/drug) (Screening & Assessment)	(*N* = *43*). Primary care providers and medical assistants, including medical assistants (*n* = 22); nurse practitioners (*n* = 9); physician assistants (*n* = 8); medical doctors (*n* = 6); doctor of osteopathy (*n* = 1); and other (*n* = 2)	Rural^a^	Qualitative (interviews, focus groups)	Yes (Knowledge to Action Framework)
Seidel [58]	2022	Germany	Hospital, Primary care	Dementia Care Management– multimodal and multiprofessional model of care for people with dementia. Comprises of management of treatment and care, medication management, and caregiver support and education (Model of Care)	(*N* = *22*). Healthcare providers, including doctors (*n* = 2); nurses and care providers (*n* = 7); palliative care providers (*n* = 1); regional networks (*n* = 1); hospitals (*n* = 4); counselling services (*n* = 4); self-help services (*n* = 3)	Regional^a^	Qualitative (interviews)	Yes (CFIR)
Shreck [59]	2020	USA	Community health	Telemental health delivered in Veteran's Health Administration through secure, real-time, interactive clinical videoconferencing systems (Digital Health Intervention)	(*N* = *9*). Psychologists	Mixed rural/regional^a^	Qualitative (document review)	No
Shulver [60]	2016	Australia	Hospital	Telehealth into mainstream healthcare services in the care of older people (Digital Health Intervention)	(*N* = *44*). Clinicians and care workers	Rural^a^	Qualitative (focus groups)	Yes (Normalisation process theory)
Stanford [61]	2019	Australia	Hospital	Patient-practitioner communication and education with Aboriginal and Torres Strait Islanders within inpatient/outpatient cardiac or coronary care units (Model of Care)	*(N* = *17).* Included nursing staff (*n* = 11); cardiologists (*n* = 5); Aboriginal health worker (*n* = 1)	Regional^a^	Qualitative (interviews)	No
Watson [62]	2022	USA	Hospital	Recovery Coach and Peer Support Initiative– aims to implement peer support services within EDs, targeting patients with OUD, linking them to treatment and services (Model of Care)	*(N not specified).* 4 ED vendors participated. Interviewees included primary leader of implementation, peers, and supervisors of peers	Rural^a^	Qualitative (interviews)	Yes (CFIR)
Wilkinson [63]	2019	Australia	Hospital	Evidence-based model of care for GDM care in dietetics, specifically Medical Nutrition Therapy (Model of Care)	(*N* = *8*). Practitioners, including GDM dietitian, site project champion, dietetics project lead, and key stakeholders	Regional^a^	Qualitative (interviews)	No

*CDC* Centres for Disease Control and Prevention, *CFIR* Consolidated Framework for Implementation Research, *ED* Emergency Department, *EPIS* Exploration, Preparation, Implementation, Sustainment, *FDHQ* Federally Qualified Health Centre, *GDM* Gestational Diabetes Mellitus, *GP* General Practitioner, *ICU* Intensive Care Unit, *OUD* Opioid Use Disorder, *PARiHS* Promoting Action on Research Implementation in Health Services, *RAC* Residential Aged Care, *TMF* Theory, Model or Framework, *USA* United States of America

^a^Indicates the article did not use a geographical classification system to define rurality

^b^Data collection approach refers to the approach used to collect barrier and facilitator data only

Most studies exclusively used qualitative methods to collect barrier/facilitator data (*n* = 33, 85%) via interviews (*n* = 18), focus groups (*n* = 6), document review (*n* = 1), or multiple qualitative approaches (*n* = 8; e.g. interviews and focus groups). Five studies (13%) used mixed-methods, while one study (3%) exclusively used a quantitative approach (survey). Less than one third of studies (*n* = 11, 28%) reported using an implementation theory, model or framework to guide an aspect of implementation; the most common was the Consolidated Framework for Implementation Research [64] (CFIR; *n* = 6, 15%).

The interventions studied were diverse. Models of care were most common (*n* = 11, 28%). Other intervention types included clinical practices/programs (*n* = 9, 23%), digital health interventions (*n* = 6, 15%), screening/assessment (*n* = 5, 13%), guidelines/recommendations (*n* = 5, 13%), and care planning (*n* = 3, 8%). Furthermore, only limited priority populations were specifically targeted in these interventions. For instance, only two studies specifically focused on Aboriginal populations [26, 61], while a single study targeted a culturally and linguistically diverse group (Latino children living in the USA) [55].

### Key barriers and facilitators to implementation

**Fig. 2:**
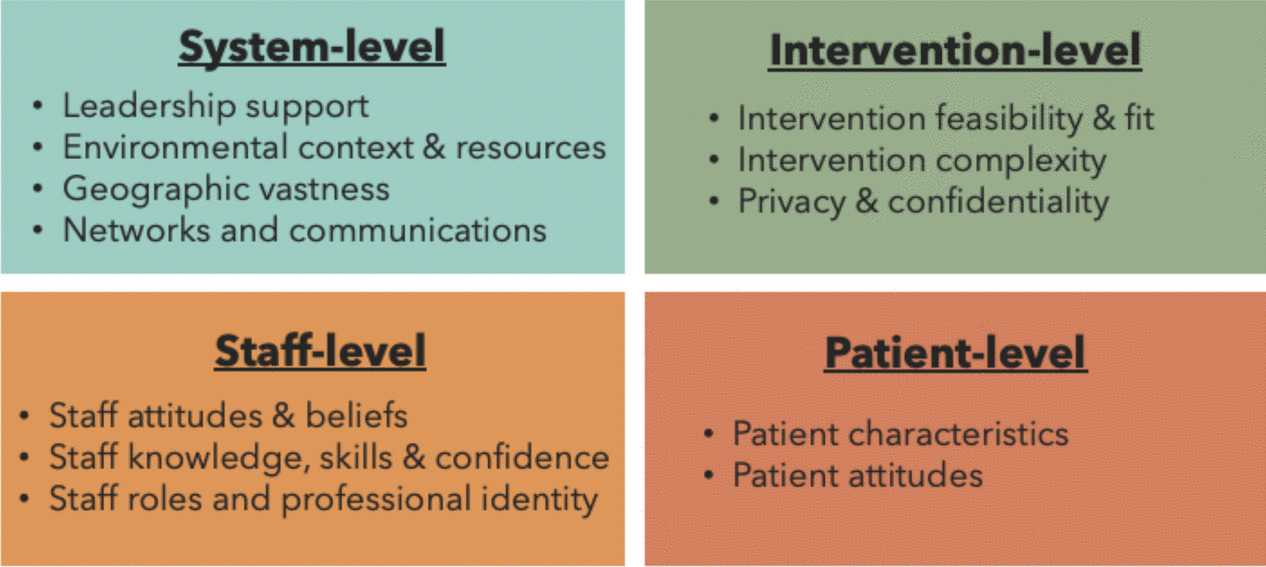
Visual depiction of themes

Key barriers and facilitators as perceived by healthcare staff, are presented below within four overarching themes: intervention-level, staff-level, patient-level, and system-level (Fig. 2). While a patient-level theme emerged from the findings, it is important to note that this theme reflects staff-reported perceptions of patient-related factors, rather than direct patient input. Specific barriers and facilitators reported in each study are detailed in Supplementary File 3.

#### Intervention-level

##### Intervention feasibility and fit

One major barrier related to the implementation of healthcare interventions in regional and rural areas was a lack of intervention feasibility. Some interventions did not align with existing workflows, systems and resources, which limited their practical implementation [29, 31]. In particular, integrating digital interventions (e.g., telehealth/those involving electronic medical records (EMR)) within organizational/systems processes posed a challenge for regional and rural healthcare professionals, as it required software compatibility across different organizations, healthcare settings and platforms [30, 49, 60]. Another perceived challenge was a lack of fit or need for a given intervention. In some instances, interventions were seen as unnecessary, incompatible or irrelevant to the needs of regional or rural healthcare providers, patients, or settings [31, 37, 58, 62]. Additionally, some healthcare professionals described having limited opportunities to implement certain interventions due to a lack of presenting patients in regional and rural areas [35, 42, 46]. While this was perceived to impact intervention feasibility and utility [35], some perceived the lack of experience with an intervention and/or specific patient population to affect staff confidence and competence [42, 46].

Conversely, the adaptability of interventions, such as cancer screening programs, that were tailored to the unique context of rural primary care practices, was determined to be a key factor that enhanced adoption [51]. Example adaptations included changing EMR functions and nurse workflows [51]. Additionally, interventions that had a high perceived need among stakeholders [49, 58], and were easily integrated into existing systems/processes [27, 58] were believed to facilitate implementation. Other enabling strategies included raising awareness and need for an intervention at a community level, including other health services and organisations [26].

##### Intervention complexity

Complexity of interventions also posed substantial challenges to their implementation in regional and rural areas. Interventions considered overly cumbersome or detailed were perceived to negatively impact the motivation of healthcare professionals [58]. Even when interventions were considered useful, their complexity was viewed as making implementation a lengthy and challenging process [58]. Rural healthcare professionals also cited the length of guidelines/recommendations as a barrier [29]. To alleviate these problems, several facilitator strategies were suggested, including the development of clear guidelines and providing healthcare professionals with summaries (or laminated copies) of clinical recommendations/checklists [29].

##### Confidentiality and privacy

Staff concerns related to the confidentiality and privacy of patients were noted as a barrier to the implementation of some healthcare interventions. This was particularly evident for interventions focused on potentially stigmatizing issues, such as mental health treatment [28] or substance use screening and/or treatment [27, 43, 57], including those that involved the sharing or storage of personal information via digital platforms [27, 30, 57]. Specifically, some staff held reservations about whether patients in rural areas would be willing to engage with an intervention due to a potential risk to their privacy [57].

#### Staff-level

##### Staff attitudes and beliefs

Several studies identified staff attitudes and beliefs as crucial factors affecting the implementation process. When healthcare professionals held positive attitudes and perceived the intervention as valuable, they were more likely to engage with and support its adoption and implementation [31, 63]. While a willing and engaged healthcare workforce was highlighted as an enabler, the lack of such a workforce was posed as a barrier [58]. Challenges in engaging staff to modify professional behaviors and adhere to intervention processes were most apparent for studies focused on the management of patients with substance use disorders in rural areas [53, 56, 62]. Additionally, staff in some studies reported that healthcare professionals held negative beliefs about the potential increase in numbers of challenging patients (e.g., people who use illicit drugs) presenting to their rural health service, which diminished healthcare professionals’ motivation to adopt the intervention [25, 56]. Conversely, personal experience was observed to be an internal motivator for some rural clinicians, leading them to support and even champion an intervention [56]. Several studies also highlighted that the identification and formal appointment of champions facilitated implementation and motivated healthcare professionals to engage with the process through positive role modelling [31, 49, 51, 53].

##### Staff knowledge, skills, and confidence

Lack of confidence among healthcare professionals emerged as a significant barrier to the implementation process, with confidence closely tied to knowledge and skill levels. Staff who reported lower knowledge and/or skills related to the intervention often felt less prepared and capable of effectively implementing interventions, which led to a reluctance to adopt new practices [39, 46]. This lack of confidence typically stemmed from limited familiarity with the intervention or inadequate training on the intervention itself and its integration into existing organizational processes [31, 33, 48]. Addressing these barriers through enhanced training and upskilling of health professionals was frequently cited as key for optimizing implementation [27, 31, 34, 35, 42, 48, 49, 57]. Participants from rural sites, who commonly reported facing workforce recruitment and retention challenges, highlighted the need for educational opportunities to be offered at orientation and repeated at intervals to ensure that new staff, including locums, are adequately informed about intervention processes [37, 46]. Supplementing training with easily accessible resources, such as manuals and checklists (digital/physical), was also suggested to facilitate improved staff knowledge and skills [31]. Additionally, providing culturally appropriate training and employing an Aboriginal Health Liaison Officer were emphasized to support staff knowledge for healthcare interventions targeting patients of Aboriginal and Torres Strait Islander descent [42, 61].

##### Staff roles and professional identity

In regional and rural settings, staff roles and professional identity influenced the implementation of healthcare interventions. Reluctance emerged when staff viewed new intervention processes as outside their primary roles or were uncertain about shifting responsibilities [57, 58]. The introduction of new roles sometimes led to staff feeling undermined, especially when role changes, and the need for them, were poorly communicated [34]. Acceptance and integration of new roles were particularly compromised if staff felt there was potential to deskill a certain profession [34, 52] (e.g., potential deskilling of rural aged care nurses with the introduction of a specialist nurse practitioner role) [34]. Conversely, a well-defined framework for staff to understand role responsibilities and clear communication channels were perceived to enhance motivation and willingness to implement [45].

#### Patient-level

##### Patient characteristics

Healthcare staff reported a range of sociodemographic characteristics to profoundly influence patients' access and interaction with healthcare interventions in regional and rural areas, which hindered the implementation ability of staff due to fewer opportunities or resources to provide effective care. In the rural context, economic constraints, including lower income levels, lack of health insurance, and increased travel costs due to geographic remoteness and limited availability of local healthcare providers, were perceived to hinder engagement with follow-up care and referral to specialist services [35, 56]. Long work hours and shift work were also highlighted as prevalent in rural communities, which limited patients' availability to attend appointments, particularly in primary care [54]. Technology-related barriers, such as inadequate telehealth infrastructure within patient homes and varying levels of technological proficiency (noted as particularly challenging among older people or those with lower educational attainment) [30] were seen to impede the effective implementation of digital health interventions [28]. To overcome barriers related to patient characteristics, the implementation of targeted strategies was recommended, including tailored patient education and support [30] and practical elements such as the provision of data SIM cards for telehealth [28].

##### Patient attitudes

Staff perceived that patient attitudes played a significant role in patients’ engagement with healthcare interventions, with a lack of engagement impacting staff opportunities to implement. One study found that rural patients' reluctance to seek healthcare was due to a cultural mindset of only addressing health issues if severe [44]. Additionally, challenges were noted in motivating patients to adopt healthier lifestyles, specifically in relation to the management of childhood obesity [35]. The reluctance of older adults to address mental health issues also emerged as a barrier, largely driven by fear of stigma in rural communities and the belief that others were more deserving of services [50]. Conversely, staff recognized that positive patient receptivity played a crucial role in facilitating the implementation of healthcare interventions. When patients engaged with and demonstrated understanding and appreciation for an intervention, it motivated health professionals to persist with their implementation efforts [53]. Furthermore, patient education and clear information materials [29], and strong patient/provider relationships [45], were seen as key factors in fostering positive patient attitudes and improving engagement with healthcare interventions in rural and regional areas.

#### System-level

##### Leadership support

Leadership support and buy-in were commonly cited as key to driving the successful implementation and sustainability of healthcare interventions in regional and rural areas. Engaged leaders were seen as essential for ensuring adequate resourcing, clearly communicating the importance of an intervention, and fostering staff buy-in [49, 53]. Leaders were also perceived to be pivotal in supporting staff attendance at meetings and education sessions [46], addressing implementation resistance within organisations [28], and securing the appropriate skill mix of personnel required for successful intervention delivery [42]. Maintaining consistent leadership across a region was also considered crucial for ensuring sustained implementation [49]. The absence of engaged leaders and internal support was also noted as a barrier in some studies [37, 42, 56, 58]. In one study, health professionals suggested this issue was more pronounced in rural hospitals, where a small number of key individuals can impact the broader staff group [56].

##### Environmental resources and context

Several barriers related to environmental resources and context were emphasized by staff in regional and rural areas. For example, budget constraints and insufficient funding for staff and services notably hindered implementation efforts [34, 38, 62, 63]. Additional challenges were highlighted concerning the reimbursement of healthcare professionals for the delivery of specific services, such as childhood obesity management [35]. High staff turnover rates and shortages in regional and rural areas were commonly perceived to exacerbate heavy workloads, time pressures, and competing priorities [33, 39, 49, 52, 53]. These factors made it challenging for staff to attend educational opportunities [46] and balance routine clinical duties with the added demands of implementing new interventions and processes [63]. In one study, researchers observed that the formal designation of a person to support staff with implementation effectively mitigated workload issues [49]. Telehealth was additionally proposed to alleviate the time pressures experienced by staff in regional and rural areas by reducing the need for travel [30].

Challenges were also noted in relation to physical and technological infrastructure. Staff reported difficulties obtaining adequate physical space [30, 63] and, in some cases, highlighted the absence of specialized care units in rural areas, such as acute stroke units [33], which hindered alignment with guidelines. Inadequate technological infrastructure, including a lack of high-speed internet, suitable hardware, and compatible software, particularly impacted the implementation of telehealth interventions [28, 30, 59]. However, investment in robust, effective, and user-friendly digital platforms was recognized to enhance the adoption of telehealth interventions [28].

##### Geographic vastness

The implementation of healthcare interventions across geographically vast areas posed notable challenges. Infrequent healthcare visits, combined with sporadic communication between patients and clinicians, as well as clinicians themselves, were cited as barriers to consistent implementation [37]. These issues were particularly highlighted by staff in rural areas, where transportation barriers and distance to local services impacted patient access and clinician adherence to guidelines [41, 54]. Furthermore, the lack of local resources and specialist services often required patients to travel long distances to urban centers for necessary care, which staff described as especially burdensome for frail older individuals without access to personal or public transportation [50].

Despite these challenges, several facilitators emerged. The use of telehealth [30] and long-standing positive patient-provider relationships [45, 47] were perceived to improve communication, coordination, and continuity of care. The sense of community in rural areas was also considered crucial, as familiarity between clinicians and patients fostered rapport and strengthened staff commitment to their communities [38, 45]. Additionally, staff noted that regional and rural services often had the flexibility to adopt new programs and practices, leading to innovative solutions tailored to their unique contexts [42, 54].

##### Networks and communication

Staff identified external communication and collaboration between services and staff as a challenge to the implementation of healthcare interventions in regional and rural areas [58, 62]. Additionally, the absence of academic affiliations was noted in one study to affect a rural hospital’s ability to access new knowledge, resources, and recruit clinicians [37]. In contrast, respected and well-connected implementers within organizations were seen as instrumental in driving implementation efforts by leveraging their social networks to identify and secure resources [37]. Informal networking in smaller communities also played a crucial role in information sharing, referrals and identifying service gaps; however, reliance on informal networks was noted to create difficulties for staff new to an area [38]. Lastly, staff in one study reported that joining a collaborative network of healthcare providers (e.g., an Accountable Care Organization) facilitated benchmarking between organizations that drove performance improvements through data comparisons [51].

## Discussion

This rapid review uniquely identified barriers and facilitators to the implementation of healthcare interventions in regional and rural healthcare services, as reported by healthcare staff working in high-income countries. The findings reveal a complex interplay of factors across four overarching themes: intervention-level, staff-level, patient-level, and system-level. While many identified barriers and facilitators align with those reported in other healthcare contexts [11, 12], this review highlights that some factors are amplified in regional and rural areas. Importantly, the review findings also emphasize that regional and rural healthcare services possess distinct strengths that can be leveraged to support the effective implementation of healthcare interventions.

Workforce shortages were found to be a critical system-level barrier and were perceived to significantly impact the implementation of healthcare interventions in regional and rural settings. Difficulties in recruiting and retaining healthcare professionals, alongside the lack of specialist services, were perceived to exacerbate workload pressures and time constraints for staff. This aligns with global concerns about the maldistribution of healthcare workers in rural areas, as highlighted by the World Health Organization (WHO) [65], where healthcare professionals are often required to cover a wide range of roles with insufficient support or resources. A shortage of specialists, such as mental healthcare providers, limits the availability of specialist care in regional and rural areas, placing an additional burden on generalists [66]. Programs like the Australian Government's Rural Health Multidisciplinary Training Program [67]. which offers healthcare students the opportunity to train in rural and remote communities, are valuable initiatives aimed at addressing these workforce gaps. This review underscores the ongoing imperative to sustain such programs to bridge the workforce divide (refer to Table 3 for summary of discussion key messages and potential actions).

**Table 3:** Discussion key messages and potential actions

Key Messages	Supporting Findings	Potential Actions
Workforce shortages and retention issues hinder implementation and sustainability	- Regional and rural healthcare providers face high workloads, limited specialist support, and high attrition, making implementation challenging	- Invest in rural workforce training and retention programs - Support multidisciplinary team models to optimize workforce distribution - Explore financial incentives for rural healthcare staff
Intervention feasibility and fit are essential for implementation success	- Rigid interventions that do not align with local workflows and resources hinder implementation	- Conduct pre-implementation needs assessments to tailor interventions to regional and rural settings - Engage healthcare leaders, frontline staff and local communities in the co-design and adaptation of interventions
Strong leadership and organizational support drive implementation	- Leadership buy-in facilitates staff engagement and access to resources	- Develop leadership training programs for regional and rural healthcare managers - Foster organizational buy-in through transparent communication - Designate implementation champions to drive change within regional and rural healthcare organizations
Digital health interventions show promise but require long-term investment	- Telehealth has the potential to improve implementation and alleviate access issues associated with geographic vastness but is limited by infrastructure gaps, digital literacy, and confidentiality concerns	- Invest in rural broadband and digital health infrastructure - Provide digital literacy training for healthcare providers and patients - Address privacy and confidentiality concerns through secure platforms
Regional and rural healthcare settings have strengths that should be leveraged to support implementation	- Strong community links, flexible healthcare services that can adapt to local needs, long-term patient-provider relationships, and informal peer networks all facilitate implementation	- Engage trusted local leaders in healthcare initiatives to foster long-term community buy-in - Foster regional collaboration networks to share resources and knowledge, building on existing community ties
Implementation science frameworks and geographic classification systems are underutilized	- Most studies did not use structured implementation frameworks to assess barriers and facilitators or use standardized geographic classification systems to define their context	- Build capacity of the rural health workforce relating to implementation science - Encourage the use of geographical classification reporting (e.g., Modified Monash Model, Rural–Urban Continuum Codes)
Limited focus on priority populations	- Few studies targeted priority populations, such as Indigenous or culturally diverse groups	- Engage priority populations in research through participatory methodologies - Apply equity-focused frameworks (e.g., Health Equity Implementation Framework)

While a patient-level theme emerged in the synthesis, these findings reflect staff-reported perceptions rather than direct patient input. Many of the identified patient-level barriers, such as attitudes toward healthcare and economic constraints, stem from broader systemic inequities rather than individual choices [68]. Factors such as lower income levels, lack of health insurance, and high travel costs for medical care disproportionately impact rural populations and limit healthcare access. Addressing these challenges requires policy and structural reforms to improve healthcare access and equity in rural settings.

Digital health interventions, particularly telehealth, were identified as potential solutions to address some of the barriers associated with environmental resources and context and geographic vastness. Telehealth reduces the need for patients and healthcare providers to travel long distances, which is particularly valuable in rural areas [69]. However, this review also identified several barriers to the effective implementation of telehealth in regional and rural settings, including inadequate digital infrastructure, limited digital literacy among healthcare staff and patients, and concerns over confidentiality and privacy. These barriers reflect findings from other studies [70, 71]. While telehealth holds promise for improving access to care, its success depends on significant investment in digital infrastructure, workforce training, and equitable access to technology, as emphasized in WHOs Global Strategy on Digital Health 2020–2025 [72]. The strategy highlights the need for robust digital health systems, increased digital literacy for healthcare providers and patients, and efforts to bridge the digital divide to ensure that telehealth benefits are accessible to all populations, particularly in low-resource settings.

At the intervention level, this review emphasizes the importance of ensuring that healthcare interventions are feasible and fit with the local context. Interventions perceived as overly complex or incompatible with existing workflows and resources were seen to have limited implementation capacity. Implementation science frameworks, such as the Implementation in Context (ICON) Framework [73] and the CFIR [64], provide guidance to implementers on the elements of context that should be considered when planning for successful implementation. Additional guidance further emphasizes the importance for interventions to be tailored to local contexts, rather than attempting to directly translate interventions developed for other settings [74]. In regional and rural settings, this may involve simplifying processes and ensuring interventions are aligned with available resources, infrastructure and staff capabilities. The adaptation of interventions and the pursuit of equity-centered implementation hinge on the authentic and meaningful participation of the community. Engaging local stakeholders, such as healthcare professionals and patients, in the co-design and adaptation of healthcare interventions is an important step that may enhance intervention relevance and acceptance, improving implementation, adoption and overall effectiveness [75]. Adaptation guidance provides structured approaches for adapting interventions to new contexts [76, 77], while practical tools like the Hexagon tool [78], and the APEASE criteria [79] can help to evaluate an intervention’s fit with the local context.

This review also highlights several strengths of regional and rural healthcare settings, that if effectively leveraged, may offset some of the identified barriers, contributing to more effective implementation of healthcare interventions. For instance, strong community relationships and flexible healthcare services that can adapt to local needs were identified as valuable assets in regional and rural areas. Long-term relationships between healthcare providers and patients in these communities can also serve as a driver to enhance patient trust and engagement with new interventions. Additionally, leadership support was seen as key to the implementation of healthcare interventions. Regional and rural healthcare services should focus on fostering strong leadership and building internal capacity to drive implementation efforts and ensure that healthcare staff are adequately supported throughout the implementation process [80].

A notable strength of this review is the comprehensive search and selection process that yielded a sizeable number of included studies published since 2000. Furthermore, data synthesis and interpretation were strengthened by an author team highly engaged in rural health research, many of whom are clinicians embedded within regional and rural healthcare services. The diversity of healthcare interventions can be seen as both a strength and a limitation of this review. While the consistency of common implementation barriers and facilitators suggests broad relevance to regional and rural healthcare settings, this diversity also limited the depth of understanding for each specific intervention type. Additionally, gaps in the available literature may limit the generalizability of the findings to certain healthcare settings or interventions not addressed in the included studies. For example, few studies were conducted outside of Australia or the USA, and aged care and community health settings were underrepresented, limiting insights into implementation factors in these contexts. Furthermore, research focusing on priority populations, such as culturally and linguistically diverse or Indigenous groups was limited. Future research should prioritise an equity-focused approach to better understand and address the unique factors influencing implementation in these populations. This may involve the use of participatory methodologies that actively engage communities, while ensuring research upholds principles of self-determination [81]. Applying equity-focused implementation frameworks, such as the Health Equity Implementation Framework [82], may also enhance the cultural responsiveness and effectiveness of healthcare interventions.

While the omission of a formal critical appraisal step is common and accepted in rapid reviews, it introduces some potential for bias, particularly regarding the strength and reliability of reported barriers and facilitators. However, the consistency of themes across studies and their alignment with existing literature [11, 12] suggest that the key findings remain robust. Future research incorporating formal risk-of-bias assessments could further enhance the reliability of synthesized evidence.

Another important finding from this review is the limited application of implementation theories, models, and frameworks in rural healthcare research. The majority of studies did not report using structured frameworks, such as the CFIR [64] or the Theoretical Domains Framework [83], to guide their assessment of barriers and facilitators. This gap highlights the need for further research to understand the feasibility, relevance, and potential impact of applying these frameworks to the implementation of healthcare interventions in non-urban settings. Future studies should also consistently apply geographical classification systems to define rurality, e.g., MMM [22] or RUCC [21]. The lack of consistent use of these classifications in the studies included in this review made it difficult to compare findings across different contexts.

Lastly, although healthcare settings differ significantly between high- and low-income countries, many of the barriers and facilitators identified in this review, such as lack of intervention fit and the importance of stakeholder engagement, have also been observed in low-income healthcare contexts [84]. Future research should explore the relevance of these factors in rural low-income healthcare settings to better understand their impact on healthcare implementation.

## Conclusion

This review provides valuable insights into the barriers and facilitators associated with implementing healthcare interventions in regional and rural settings, as perceived by healthcare staff in high-income countries. Addressing the challenges faced in these contexts while leveraging their inherent strengths has the potential to significantly enhance implementation outcomes and contribute to reducing the health disparity gap between rural and metropolitan populations. Policymakers, healthcare leaders, and researchers tasked with designing and implementing healthcare interventions in regional and rural areas should systematically consider barriers and facilitators across the four overarching themes: intervention-, staff-, patient-, and system-level. Conducting context-specific assessments that utilize established implementation frameworks will enable stakeholders to design tailored implementation strategies that have the capacity to effectively support healthcare staff and optimize service delivery.

## Supplementary Information

Supplementary Material 1.

Supplementary Material 2.

Supplementary Material 3.

## Data Availability

The datasets used and/or analysed during the current study are available from the corresponding author on reasonable request.

## Abbreviations

EBI: Evidence-based intervention
PRISMA: Preferred Reporting Items for Systematic reviews and Meta-Analysis
RUCC: Rural-Urban Continuum Codes
MMM: Modified Monash Model
USA: United States of America
CFIR: Consolidated Framework for Implementation Research
EMR: Electronic Medical Records
WHO: World Health Organization
ICON: Implementation in Context
