# Supplementary material for: Staff-reported barriers and facilitators to the implementation of healthcare interventions within regional and rural areas: a rapid review
Source: BMC Health Serv Res. 2025 Mar 4;25:331. doi: 10.1186/s12913-025-12480-8 (PMC11877690; doi:10.1186/s12913-025-12480-8)
Supplement: Supplementary file 2 — Supplementary Material 2. [file 12913_2025_12480_MOESM2_ESM.pdf]

**Supplementary File 2: Search Strategy**

**Medline Complete**

1. (MH "Rural Health Services+") OR (MH "Rural Health")
2. (AB rural OR TI rural) OR (AB regional OR TI regional)
3. S1 OR S2
4. (MH "Implementation Science")
5. (MH "Diffusion of Innovation")
6. (MH "Delivery of Health Care")
7. (AB implement* OR TI implement*) OR (AB translat* OR TI translat*) OR (AB provision OR TI provision) OR (AB provide OR TI provide)
8. S4 OR S5 OR S6 OR S7
9. (MH "Health Services")
10. (AB hospital OR TI hospital) OR (AB "acute care" OR TI "acute care") OR (AB "sub*acute care" OR TI "sub*cute care") OR (AB "aged care" OR TI "aged care") OR (AB ag?ing OR TI ag?ing) OR (AB "primary care" OR TI "primary care") OR (AB "primary health care" OR TI "primary health care") OR (AB "health service*" OR TI "health service*") OR (AB “health care service*” OR TI “health care service*”) OR (AB “healthcare service*” OR TI “healthcare service*”) OR (AB "community health OR TI "community health") OR (AB "hospital service*" OR TI "hospital service*") OR (AB "hospital program*" OR TI "hospital program*) OR (AB "tertiary service*" OR TI "tertiary service*") OR (AB "health service* research) OR (TI "health service* research")
11. S9 OR S10
12. ((AB facilitat* OR TI facilitat*) OR (AB barrier* OR TI barrier*) OR (AB challeng* OR TI challeng*) OR (AB "barrier analysis" OR TI "barrier analysis") OR (AB "process analysis" OR TI "process analysis") OR (AB enabl* OR TI enabl*) OR (AB "change agent" OR TI "change agent") OR ( AB “process evaluation” OR TI “process evaluation”) OR (AB “motivator*” OR TI “motivator*”))
13. S3 AND S8 AND S11 AND S12
14. S13. Full Text; Date of Publication: 20000101-20231231; English Language

**CINAHL complete (EBSCOhost)**

(Same as Medline search) Full Text; Published Date: 20000101-20231231; English Language; Research Article; Exclude MEDLINE records.

**Embase**

1. 'rural health care'/exp
2. rural:ab,ti OR regional:ab,ti
3. #1 OR #2
4. 'implementation science'/exp
5. 'diffusion of innovation'/exp
6. 'health care delivery'/de
7. implement*:ab,ti OR tranlat*:ab,ti OR provision:ab,ti OR provide:ab,ti
8. #4 OR #5 OR #6 OR #7
9. 'health service'/de
10. hospital:ab,ti OR 'acute care':ab,ti OR 'sub*acute care':ab,ti OR 'aged care':ab,ti OR ag?ing:ab,ti OR 'primary care':ab,ti OR 'primary health care':ab,ti OR 'health service*':ab,ti OR 'health care service*':ab,ti OR 'healthcare service*':ab,ti OR 'community health':ab,ti OR

'hospital service*':ab,ti OR 'hospital program*':ab,ti OR 'tertiary service*':ab,ti OR 'health service* research':ab,ti
11. #9 OR #10
12. facilitat*:ab,ti OR barrier*:ab,ti OR challeng*:ab,ti OR 'barrier analysis':ab,ti OR 'process analysis':ab,ti OR enabl*:ab,ti OR 'change agent':ab,ti OR 'process evaluation':ab,ti OR motivator*:ab,ti
13. #3 AND #8 AND #11 AND #12
14. #13 AND (2000:py OR 2001:py OR 2002:py OR 2003:py OR 2004:py OR 2005:py OR 2006:py OR 2007:py OR 2008:py OR 2009:py OR 2010:py OR 2011:py OR 2012:py OR 2013:py OR 2014:py OR 2015:py OR 2016:py OR 2017:py OR 2018:py OR 2019:py OR 2020:py OR 2021:py OR 2022:py OR 2023:py) AND [embase]/lim NOT ([embase]/lim AND [medline]/lim) AND ('article'/it OR 'article in press'/it)
15. #14 AND [english]/lim

**APA PsycInfo**

1. DE “rural health”
2. (AB rural OR TI rural) OR (AB regional OR TI regional)
3. S1 OR S2
4. DE "Health Care Delivery"
5. (AB implement* OR TI implement*) OR (AB translat* OR TI translat*) OR (AB provision OR TI provision)) OR (AB provide OR TI provide)
6. S4 OR S5
7. DE "Health Care Services"
8. (AB hospital OR TI hospital) OR (AB "acute care" OR TI "acute care") OR (AB "sub*acute care" OR TI "sub*acute care") OR (AB "aged care" OR TI "aged care") OR (AB ag?ing OR TI ag?ing) OR (AB "primary care" OR TI "primary care") OR (AB "primary health care" OR TI "primary health care") OR (AB "health service*" OR TI "health service*") OR (AB “health care service*” OR TI “health care service*”) OR (AB “healthcare service*” OR TI “healthcare service*”) OR (AB "community health OR TI "community health") OR (AB "hospital service*" OR TI "hospital service*") OR (AB "hospital program*" OR TI "hospital program*) OR (AB "tertiary service*" OR TI "tertiary service*") OR (AB "health service* research" OR TI "health service* research")
9. S7 OR S8
10. ((AB facilitat* OR TI facilitat*) OR (AB barrier* OR TI barrier*) OR (AB challeng* OR TI challeng*) OR (AB "barrier analysis" OR TI "barrier analysis") OR (AB "process analysis" OR TI "process analysis") OR (AB enabl* OR TI enabl*) OR (AB "change agent" OR TI "change agent") OR (AB “process evaluation” OR TI “process evaluation”) OR (AB “motivator*” OR TI “motivator*”))
11. S3 AND S7 AND S9 AND S10
12. S11 Limiters - Linked Full Text; Published Date: 20000101-20231231; English
