# Supplementary material for: Staff-reported barriers and facilitators to the implementation of healthcare interventions within regional and rural areas: a rapid review
Source: BMC Health Serv Res. 2025 Mar 4;25:331. doi: 10.1186/s12913-025-12480-8 (PMC11877690; doi:10.1186/s12913-025-12480-8)
Supplement: Supplementary file 3 — Supplementary Material 3. [file 12913_2025_12480_MOESM3_ESM.pdf]

Supplementary File 3. Summary of key barriers and facilitators reported in each included study (N=39)

Author	Title	Key barriers	Key facilitators
Andrilla[1]	Barriers rural physicians face prescribing buprenorphine for opioid use disorder	• Time limitations<br>• Lack of patient need<br>• Financial/reimbursement concerns<br>• Resistance from practice partner<br>• Lack of specialty backup for complex problems<br>• Lack of confidence in ability to manage opioid use disorder<br>• Lack of available mental health or psychosocial support services<br>• Attraction of drug users to your practice<br>• Diversion or misuse of medication concerns	
Beks[2]	An Aboriginal Community-Controlled Health Organization model of service delivery: Qualitative process evaluation of the Tulku wan Wininn mobile clinic	• Technical issues<br>• Logistical issues<br>• Operational issues	• Raising awareness of intervention at a community level<br>• Mobile Clinic Coordinator<br>• Having accessible and permanent GP services<br>• Targeted strategies to expand the reach of mobile clinics (intervention) to Aboriginal and/or Torres Strait Islander Peoples<br>• Modifying physical space to improve accessibility<br>• Expanding scope of intervention/delivering more clinical services<br>• Comfort and preparedness discussing and treating substance use
Berends[3]	Implementation effectiveness of an alcohol-screening and intervention project at two hospitals in regional Victoria, Australia	• Staff turnover<br>• Staff capacity<br>• Concerns about patient privacy<br>• Competing demands on staff time	• Provision of training<br>• Leadership attitudes<br>• Capacity of project worker<br>• Project integration

		• Policies	
Chatterton[4]	Telehealth service delivery in an Australian regional mental health service during COVID-19: A mixed methods analysis	• Inadequate IT infrastructure<br>• Engaging with telehealth intervention was complex<br>• Provider perceptions of consumer's attitudes to telehealth	• Early positive and successful experiences of telehealth<br>• Investment in telehealth infrastructure<br>• Development of clear guidelines<br>• Leadership in telehealth implementation<br>• Mechanisms to share knowledge
Daugherty[5]	Rural primary care providers' experience and usage of clinical recommendations in the CDC pediatric mild traumatic brain injury guideline: A qualitative study	• Providers lack access to specialists<br>• Recommendations are too long<br>• Not all the recommendations are feasible in rural areas	• Summarise clinical recommendations<br>• Link recommendations to clinical tools<br>• More educational tools for the public about mTBI (intervention)
DeHart[6]	Benefits and challenges of implementing telehealth in rural settings: A mixed-methods study of behavioral medicine providers	• Organisational capacity<br>• Sustainability<br>• Patient skills and comfort<br>• Provider knowledge and skills	• Increased inter- and intra-agency coordination<br>• Clinician travel time<br>• Geographic access<br>• Transportation (patient)<br>• Critical incidents/natural disasters<br>• Patient time
Delaforce[7]	Creating an implementation enhancement plan for a digital patient fall prevention platform using the CFIR-ERIC approach: A qualitative study	• Access to knowledge and information<br>• Patient needs and resources<br>• Available resources<br>• Adaptability<br>• Design quality and packaging<br>• Compatibility	• Access to knowledge and information<br>• Patient needs and resources<br>• Formally appointed internal implementation leaders<br>• Cosmopolitanism<br>• Knowledge and beliefs about the intervention<br>• Relative advantage
Druskin[8]	The dissemination of parent-child interaction therapy in West Virginia during the opioid epidemic and COVID-19 pandemic: A qualitative study	• Patient technology barriers<br>• Low family/patient resources<br>• Telehealth format impairs therapist-family (patient) connection	• Agency support
Dwyer[9]	Health care providers perceptions of factors that influence the provision of acute stroke care in urban and rural settings: A qualitative study	• Infrastructure<br>• Staffing/workload constraints<br>• Varied styles<br>• Protocol/procedures<br>• Staff reluctance<br>• Expert involvement	

Ervin[10]	Implementation of an older person's nurse practitioner in rural aged care in Victoria, Australia: A qualitative study	• Funding<br>• Staff engagement<br>• Social/professional roles<br>• Knowledge<br>• Legislation	• Reflexive monitoring<br>• Knowledge<br>• Legislation
Findholt[11]	Perceived barriers, resources, and training needs of rural primary care providers relevant to the management of childhood obesity	• Time constraints<br>• Reimbursement<br>• Limited opportunities<br>• Lack of specialists/services<br>• Cost to families<br>• Patient motivation/lifestyle<br>• Issue sensitivity<br>• Knowledge	• Role establishment<br>• Knowledge
Fletcher[12]	Rural health professionals' experiences in implementing advance care planning: A focus group study	• Lack of time<br>• Lack of confidence<br>• Lack of patient engagement<br>• The desire to 'preserve home'<br>• Complexity of advance care planning	
Harrod[13]	Unique factors rural Veterans' Affairs hospitals face when implementing health care-associated infection prevention initiatives	• Staff recruitment and retention<br>• Lack of internal support<br>• Absence of academic affiliations<br>• System-wide mandates<br>• Geographic distance of sites	• External relationships/resources<br>• Telemedicine<br>• Respected/socially connected implementers
Henderson[14]	Regional responses to the challenge of delivering integrated care to older people with mental health problems in rural Australia	• Access to specialists<br>• Change of federal government<br>• Fragmentation of service delivery<br>• Funding models<br>• Bureaucratisation and centralisation<br>• Role of informal networking	• Sense of community<br>• Self-sufficiency<br>• Role of informal networking
Hill[15]	Assessing adherence and exploring barriers to provision of prescribed texture modifications for dysphagia in a residential aged care facility in rural Australia	• Communication processes<br>• Knowledge and confidence applying knowledge<br>• Time pressures on staff<br>• Staffing experience and availability<br>• Balancing preferences/ risk<br>• Workplace culture<br>• Limited resources	
Howland[16]	Psychiatrist and psychologist	• Difficulty mobilising community	• Appointing a "point person" to assist

	experiences with telehealth and remote collaborative care in primary care: A qualitative study	resources in emergency situations<br>• Logistical challenges in clinics<br>• Staff engagement (with intervention)<br>• Low clinic-level investment/resources	with clinic communication<br>• Staff engagement (with intervention)<br>• Close communication with staff
Khoong[17]	Rural, suburban, and urban differences in factors that impact physician adherence to clinical preventive service guidelines	• Distance<br>• Patient resistance<br>• Resources<br>• Access to specialists<br>• Referred services	• Health professional prioritisation
Kilcullen[18]	Palliative care in the neonatal unit: neonatal nursing staff perceptions of facilitators and barriers in a regional tertiary nursery	• Time pressures on families<br>• Availability of specialist teams<br>• Fewer experiences<br>• Technology engagement<br>• Staff – increased need to support families<br>• Leadership<br>• Clinical knowledge/education	• Creative care<br>• Cultural awareness and cultural support for staff and families<br>• Leadership<br>• Clinical knowledge/education
Kirstman-Valente[19]	Barriers to implementing a cannabis focused SBIRT in adolescent primary care	• Time limitations<br>• Billing/reimbursement<br>• Confidentiality issues<br>• Resource constraints and training needs<br>• More concern for other substances (providers' lack of prioritisation of intervention)<br>• Patient-parent ambivalence to health issue (cannabis use)<br>• Provider ambivalence toward cannabis use (health issue)	
Kruse-Diehr[20]	Building cancer prevention and control research capacity in rural Appalachian Kentucky primary care clinics during COVID-19: Development and adaptation of a multilevel colorectal cancer screening project	• Patients' feelings of fatalism<br>• Not being viewed as a priority by patients<br>• Electronic health record limitations	
Lam[21]	Current practices, barriers and enablers for advance care planning among healthcare workers of aged care facilities in western New South Wales, Australia		• Pre-emptive discussions of advance care planning (intervention)<br>• Having a strong relationship with

			patients and their families<br>• Having a framework for healthcare workers<br>• Training and upskilling<br>• Sense of community in rural settings
Lillebuen[22]	Facilitators and barriers to care in rural emergency departments in Alberta for patients on peritoneal dialysis (PD): An interpretive descriptive study	• Staff motivation/buy-in<br>• Infrequent exposure<br>• Physician support<br>• Education<br>• Resources	• Management support<br>• Patient knowledge/skill<br>• Education<br>• Resources
Littlewood[23]	Advance care planning in rural New South Wales from the perspective of general practice registrars and recently fellowed general practitioners	• Lack of patient understanding of advanced care planning/doctor dependent uptake<br>• Demands on doctor's time<br>• Lack of specialist support in rural areas	• Long-standing relationships in the rural setting<br>• Patient's right to choose
Maxwell[24]	Staff experiences of a reablement approach to care for older people in a regional Australian community: A qualitative study	• Lack of human resources<br>• Education and training	• Valuing client-centred care<br>• Communication between direct care staff and care coordinators (partnerships in care)<br>• Education and training
Morgan[25]	Barriers and facilitators to development and implementation of a rural primary health care intervention for dementia: a process evaluation	• External policy and incentives<br>• Networks and communications<br>• Compatibility with existing workflows and processes (implementation climate sub-construct)<br>• Available resources (readiness for implementation subconstruct)<br>• Innovation sustainability	• Needs and resources of those served by the innovation<br>• Tension for change (implementation climate sub-construct)<br>• Leadership engagement (readiness for implementation sub-construct)<br>• Access to knowledge and information<br>• Champions<br>• External policy and incentives<br>• Networks and communications<br>• Compatibility with existing workflows and processes (implementation climate sub-construct)<br>• Available resources (readiness for implementation subconstruct)<br>• Innovation sustainability
Muir-	Service provision for older people with	• Patient attitudes	• Knowledge about services

Cochrane[26]	mental health problems in a rural area of Australia	• Health professional attitudes<br>• Service availability<br>• Service appropriateness<br>• Transport<br>• Collaboration between health care organisations<br>• Knowledge about services	
Nelson-Brantley[27]	Implementation of cancer screening in rural primary care practices after joining an accountable care organisation: A multiple case study		• Joining an accountable care organisation<br>• Benchmarking<br>• Integrating screening into electronic health record<br>• Champions<br>• Adaptability to local context
Paliadelis[28]	Implementing family-centred care: an exploration of the beliefs and practices of paediatric nurses	• Time constraints<br>• Staff shortages/heavy workloads<br>• Professional role/identity	• Professional role/identity
Parchman[29]	Barriers and facilitators to implementing changes in opioid prescribing in rural primary care clinics	• Competing demands/priorities<br>• Clinician attitudes/beliefs<br>• Inadequate data systems<br>• Lack of local resources	• Supportive leadership<br>• Patient receptivity<br>• External support<br>• Work-life stress<br>• External pressure<br>• Desire to help patients/community
Porter[30]	Examining the feasibility and characteristics of realistic weight management support for patients: Focus groups with rural, micropolitan, and metropolitan primary care providers	• Geographic distance<br>• Transportation<br>• Patient work schedule	• Service independence
Rosado[31]	From planning to implementation: Developing an ACE screening protocol in a rural integrated primary care clinic serving Latino children	• Time constraints<br>• Provider knowledge<br>• Patient literacy<br>• Family attitudes<br>• Resources	
Rosenberg[32]	Barriers and facilitators associated with establishment of emergency department-initiated buprenorphine for opioid use disorder in rural Maine	• Hospital membership<br>• Fear of overcrowding<br>• Insufficient outreach (to patient community)<br>• Lack of follow-up	• Personal experience as internal motivator<br>• Peer example<br>• Hospital membership<br>• Peer mentorship and collaboration

Saunders[33]	Screening for substance use in rural primary care: A qualitative study of providers and patients	• Patient concerns about the consequences of disclosing substance use<br>• Electronic health record privacy concerns<br>• Lack of time	• Education and training
Seidel[34]	Adapting a dementia care management intervention for regional implementation: a theory-based participatory barrier analysis	• Patient needs<br>• Intersectoral networking and communication between healthcare providers<br>• Engaging stakeholders (leaders, champions etc.)<br>• Complexity of the dementia care management (DeCM) intervention<br>• Attitude and behaviour of healthcare providers	• Patient needs<br>• Intersectoral networking and communication between healthcare providers<br>• Willingness of stakeholders to implement intervention<br>• High perceived need for DeCM<br>• Fit between DeCM and existing networks, internal processes and infrastructure<br>• Availability of motivated and flexible stakeholders<br>• Resources available for implementation
Shreck[35]	Barriers and facilitators to implementing a US Department of Veterans Affairs Telemental Health (TMH) program for rural veterans	• "Network model"<br>• Provision of services<br>• Therapeutic relationship impact<br>• Evolution of technology<br>• Use of materials<br>• Integration of Hub missions<br>• Procedural set-up of services	• Provision of services<br>• Therapeutic relationship impact<br>• Evolution of technology<br>• Use of materials<br>• Integration of Hub missions<br>• Procedural set-up of services
Shulver[36]	'Massive potential' or 'safety risk'? Health worker views on telehealth in the care of older people and implications for successful normalization	• Contextual integration	• Workability of telehealth
Stanford[37]	Better cardiac care: Health professional's perspectives of the barriers and enablers of health communication and education with patients of Aboriginal and Torres Strait Islander descent	• Lack of time<br>• Limited availability of culturally appropriate services and resources<br>• Conflicting imperatives of organisational structures	• Having an Aboriginal Liaison Officer<br>• Consistent reinforcement of education for patients (of Aboriginal and Torres Strait Islander descent)<br>• Better co-ordination of care and collaboration amongst the multidisciplinary team<br>• Further training<br>• Having educational materials or

			resources to give to patients
Watson[38]	Identifying unique barriers to implementing rural emergency department-based peer services for opioid use disorder through qualitative comparison with urban sites	• Cost<br>• Needs of target/rural population<br>• External relational climate<br>• Poor local infrastructure<br>• Networks and communication amongst inner setting actors<br>• Poor implementation climate<br>• Knowledge and beliefs of staff members<br>• Engaging health providers	• Engaging health providers
Wilkinson[39]	Implementing a best-practice model of gestational diabetes mellitus care in dietetics: A qualitative study	• Resourcing constraints<br>• Uncertainty about project/intervention logistics<br>• Juggling clinical and project workloads	• A strong focus on team communication and support<br>• Management buy-in<br>• Escalating resource issues to statewide allied health management<br>• Positive attitude

References:

1. Andrilla CHA, Coulthard C, Larson EH. Barriers Rural Physicians Face Prescribing Buprenorphine for Opioid Use Disorder. *Ann Fam Med.* Jul 2017;15(4):359-362.
2. Beks H, Mitchell F, Charles JA, McNamara KP, Versace VL. An Aboriginal Community-Controlled Health Organization model of service delivery: qualitative process evaluation of the Tulku wan Wininn mobile clinic. *International Journal for Equity in Health.* 2022/11/16 2022;21(1):163.
3. Berends L, Roberts B. Implementation Effectiveness of an Alcohol-Screening and Intervention Project at Two Hospitals in Regional Victoria, Australia. *Contemporary Drug Problems.* 2012;39(2):289-309.
4. Chatterton ML, Marangu E, Clancy EM, et al. Telehealth service delivery in an Australian regional mental health service during COVID-19: a mixed methods analysis. *Int J Ment Health Syst.* Aug 19 2022;16(1):43.
5. Daugherty J, Waltzman D, Popat S, Groenendaal AH, Cherney M, Knudson A. Rural Primary Care Providers' Experience and Usage of Clinical Recommendations in the CDC Pediatric Mild Traumatic Brain Injury Guideline: A Qualitative Study. *J Rural Health.* Jun 2021;37(3):487-494.
6. DeHart D, King LB, Iachini AL, Browne T, Reitmeier M. Benefits and Challenges of Implementing Telehealth in Rural Settings: A Mixed-Methods Study of Behavioral Medicine Providers. *Health & Social Work.* 2021;47(1):7-18.
7. Delaforce A, Li J, Grujovski M, et al. Creating an Implementation Enhancement Plan for a Digital Patient Fall Prevention Platform Using the CFIR-ERIC Approach: A Qualitative Study. *Int J Environ Res Public Health.* Feb 21 2023;20(5).

8. Druskin LR, Han RC, Phillips ST, et al. The Dissemination of Parent-Child Interaction Therapy in West Virginia during the Opioid Epidemic and COVID-19 Pandemic: A Qualitative Study. *Int J Environ Res Public Health.* Nov 16 2022;19(22).
9. Dwyer M, Peterson GM, Gall S, Francis K, Ford KM. Health care providers' perceptions of factors that influence the provision of acute stroke care in urban and rural settings: A qualitative study. *SAGE Open Med.* 2020;8:2050312120921088.
10. Ervin K, Reid C, Moran A, Opie C, Haines H. Implementation of an older person's nurse practitioner in rural aged care in Victoria, Australia: a qualitative study. *Hum Resour Health.* Nov 1 2019;17(1):80.
11. Findholt NE, Davis MM, Michael YL. Perceived Barriers, Resources, and Training Needs of Rural Primary Care Providers Relevant to the Management of Childhood Obesity. *The Journal of Rural Health.* 2013;29(s1):s17-s24.
12. Fletcher S, Sinclair C, Rhee J, Goh D, Auret K. Rural health professionals' experiences in implementing advance care planning: a focus group study. *Aust J Prim Health.* Nov 2016;22(5):423-427.
13. Harrod M, Manojlovich M, Kowalski CP, Saint S, Krein SL. Unique factors rural Veterans' Affairs hospitals face when implementing health care-associated infection prevention initiatives. *J Rural Health.* Winter 2014;30(1):17-26.
14. Henderson J, Dawson S, Fuller J, et al. Regional responses to the challenge of delivering integrated care to older people with mental health problems in rural Australia. *Aging Ment Health.* Aug 2018;22(8):1025-1031.
15. Hill C, Clapham RP, Buccheri A, Field M, Wong Shee A, Alston L. Assessing adherence and exploring barriers to provision of prescribed texture modifications for dysphagia in a residential aged care facility in rural Australia. *Int J Speech Lang Pathol.* Feb 2022;24(1):67-76.
16. Howland M, Tennant M, Bowen DJ, et al. Psychiatrist and Psychologist Experiences with Telehealth and Remote Collaborative Care in Primary Care: A Qualitative Study. *J Rural Health.* Sep 2021;37(4):780-787.
17. Khoong EC, Gibbert WS, Garbutt JM, Sumner W, Brownson RC. Rural, suburban, and urban differences in factors that impact physician adherence to clinical preventive service guidelines. *J Rural Health.* Winter 2014;30(1):7-16.
18. Kilcullen M, Ireland S. Palliative care in the neonatal unit: neonatal nursing staff perceptions of facilitators and barriers in a regional tertiary nursery. *BMC Palliat Care.* May 11 2017;16(1):32.
19. Kristman-Valente AN, McCarty CA, Walker DD, Walker-Harding L. Barriers to Implementing a Cannabis Focused SBIRT in Adolescent Primary Care. *Subst Abuse.* 2022;16:11782218221111837.
20. Kruse-Diehr AJ, Dignan M, Cromo M, et al. Building Cancer Prevention and Control Research Capacity in Rural Appalachian Kentucky Primary Care Clinics During COVID-19: Development and Adaptation of a Multilevel Colorectal Cancer Screening Project. *J Cancer Educ.* Oct 2022;37(5):1407-1413.
21. Lam L, Ansari AS, Baquir PJ, Chowdhury N, Tran K, Bailey J. Current practices, barriers and enablers for advance care planning among healthcare workers of aged care facilities in western New South Wales, Australia. *Rural Remote Health.* Nov 2018;18(4):4714.
22. Lillebuen L, Schick-Makaroff K, Thompson S, Molzahn A. Facilitators and Barriers to Care in Rural Emergency Departments in Alberta for Patients on Peritoneal Dialysis (PD): An Interpretive Descriptive Study. *Can J Kidney Health Dis.* 2020;7:2054358120970098.
23. Littlewood J, Hinchcliff R, Lo W, Rhee J. Advance care planning in rural New South Wales from the perspective of general practice registrars and recently fellowed general practitioners. *Aust J Rural Health.* Oct 2019;27(5):398-404.
24. Maxwell H, Bramble M, Prior SJ, et al. Staff experiences of a reablement approach to care for older people in a regional Australian community: A qualitative study. *Health Soc Care Community.* May 2021;29(3):685-693.

25. Morgan D, Kosteniuk J, O’Connell ME, et al. Barriers and facilitators to development and implementation of a rural primary health care intervention for dementia: a process evaluation. *BMC Health Services Research.* 2019/10/17 2019;19(1):709.

26. Muir-Cochrane E, O'Kane D, Barkway P, Oster C, Fuller J. Service provision for older people with mental health problems in a rural area of Australia. *Aging Ment Health.* 2014;18(6):759-766.

27. Nelson-Brantley H, Ellerbeck EF, McCrea-Robertson S, et al. Implementation of cancer screening in rural primary care practices after joining an accountable care organisation: a multiple case study. *Fam Med Community Health.* Dec 2021;9(4).

28. Paliadelis P, Cruickshank M, Wainohu D, Winskill R, Stevens H. Implementing family-centred care: an exploration of the beliefs and practices of paediatric nurses. *Aust J Adv Nurs.* Sep-Nov 2005;23(1):31-36.

29. Parchman ML, Ike B, Osterhage KP, Baldwin LM, Stephens KA, Sutton S. Barriers and facilitators to implementing changes in opioid prescribing in rural primary care clinics. *J Clin Transl Sci.* Jan 10 2020;4(5):425-430.

30. Porter GC, Schwab R, Hill JL, et al. Examining the feasibility and characteristics of realistic weight management support for patients: Focus groups with rural, micropolitan, and metropolitan primary care providers. *Prev Med Rep.* Sep 2021;23:101390.

31. Rosado JI, Reyes E, Montgomery J, Wang Y, Malloy C, Simpson-O'Reggio AM. From planning to implementation: Developing an ACE screening protocol in a rural integrated primary care clinic serving Latino children. *Clinical Practice in Pediatric Psychology.* 2024;12(1):36-47.

32. Rosenberg NK, Hill AB, Johnsky L, Wiegn D, Merchant RC. Barriers and facilitators associated with establishment of emergency department-initiated buprenorphine for opioid use disorder in rural Maine. *J Rural Health.* Jun 2022;38(3):612-619.

33. Saunders EC, Moore SK, Gardner T, et al. Screening for Substance Use in Rural Primary Care: a Qualitative Study of Providers and Patients. *J Gen Intern Med.* Dec 2019;34(12):2824-2832.

34. Seidel K, Quasdorf T, Haberstroh J, Thyrian JR. Adapting a Dementia Care Management Intervention for Regional Implementation: A Theory-Based Participatory Barrier Analysis. *Int J Environ Res Public Health.* Apr 30 2022;19(9).

35. Shreck E, Nehrig N, Schneider JA, et al. Barriers and facilitators to implementing a U.S. Department of Veterans Affairs Telemental Health (TMH) program for rural veterans. *Journal of Rural Mental Health.* 2020;44(1):1-15.

36. Shulver W, Killington M, Crotty M. 'Massive potential' or 'safety risk'? Health worker views on telehealth in the care of older people and implications for successful normalization. *BMC Med Inform Decis Mak.* Oct 12 2016;16(1):131.

37. Stanford J, Charlton K, McMahon A-T, Winch S. Better cardiac care: health professional’s perspectives of the barriers and enablers of health communication and education with patients of Aboriginal and Torres Strait Islander descent. *BMC Health Services Research.* 2019/02/07 2019;19(1):106.

38. Watson DP, Staton MD, Gastala N. Identifying unique barriers to implementing rural emergency department-based peer services for opioid use disorder through qualitative comparison with urban sites. *Addiction Science & Clinical Practice.* 2022/07/28 2022;17(1):41.

39. Wilkinson SA, O’Brien M, McCray S, Harvey D. Implementing a best-practice model of gestational diabetes mellitus care in dietetics: a qualitative study. *BMC Health Services Research.* 2019/02/14 2019;19(1):122.
